# Comparative Study of Different Operation Modes of Microbial Fuel Cells Treating Food Residue Biomass

**DOI:** 10.3390/molecules26133987

**Published:** 2021-06-29

**Authors:** Asimina Tremouli, Theofilos Kamperidis, Gerasimos Lyberatos

**Affiliations:** 1 School of Chemical Engineering, National Technical University of Athens, Heroon Polytechniou 9, Zografou, 15780 Athens, Greece; kamp.theo@gmail.com (T.K.); lyberatos@chemeng.ntua.gr (G.L.); 2 Institute of Chemical Engineering Sciences (ICE-HT), Stadiou Str., Platani, 26504 Patras, Greece

**Keywords:** household food waste, microbial fuels cells, bioelectricity, stacks, FORBI

## Abstract

Four multiple air–cathode microbial fuel cells (MFCs) were developed under the scope of using extracts from fermentable household food waste (FORBI) for the production of bioelectricity. The operation of the MFCs was assessed in batch mode, considering each cell individually. Τhe chemical oxygen demand (COD) efficiency was relatively high in all cases (>85% for all batch cycles) while the electricity yield was 20 mJ/gCOD/L of extract solution. The four units were then electrically connected as a stack, both in series and in parallel, and were operated continuously. Approximately 62% COD consumption was obtained in continuous stack operation operated in series and 67% when operated in parallel. The electricity yield of the stack was 2.6 mJ/gCOD/L of extract solution when operated continuously in series and 0.7 mJ/gCOD/L when operated continuously in parallel.

## 1. Introduction

The increase in global population, accompanied with the increase in energy needs, environmental pollution and the depletion of natural resources, makes it more than a necessity to change the world’s approach towards waste [1]. In this direction, waste is nowadays considered more as a valuable resource than just a material to dispose. Using appropriate technologies, waste, whether pretreated or not, can be transformed into value-added products.

Food waste (FW) is a global concern since a tremendous amount of it is generated every year. In the European Union alone, 88 million tons of FW is produced annually, while in the United States [2], approximately 103 million tons of FW is generated yearly, corresponding to the 30–40% of the total food supply [3]. Paradoxically, 821 million people are malnourished at the same time, which shows that wasting food is a substantial waste of resources since land, water and energy are required for food production, processing and distribution. At the same time, food loss is responsible for 8% of the total global GHG emissions [4,5]. Due to FW composition (high water content, complex organic matter and other nutrients), FW is easily biodegraded by aerobic and/or anaerobic microorganisms while emitting odors and spreading diseases [6,7]. As a solution to the traditional FW treatments (incineration, landfilling, composting) that contribute to the environmental pollution, FW can be considered as a valuable biomass resource from which biodiesel, bioethanol, biohydrogen, biomethane, butanol, biosurfactants, bio-plastics, organic fertilizers and bioelectricity can be produced using various bioprocesses [8].

Microbial fuel cell (MFC) is an emerging biotechnology which is very promising towards FW treatment MFCs are bioelectrochemical systems that spontaneously generate electricity while treating wastewater, through the metabolic activity of microorganisms [9,10,11]. This unique feature of the technology has attracted scientific interest and a vast range of organic wastes/resources have been treated in an MFC with parallel bioelectricity production [12]. Although a lot of effort has been made to develop MFC technology, these systems still generate low power outputs. Further to the thermodynamic limitations of these units, different voltage losses contribute to low levels of bioelectricity production. These losses may originate from redox reactions, the MFC design, the construction materials, etc. [13,14,15]. In order to overcome these limitations, various designs and materials have been proposed [16,17,18]. Moreover, stacking multiple MFC units together is suggested as a solution to improve power outputs towards the practical implementation of MFC [19,20,21]. However, stacking multiple units together carries its own challenges, such as the voltage reversal phenomenon. Voltage reversal occurs when one or more cells underperform in comparison to other cells within the stack, causing a total failure of the system’s performance [22,23].

In the European Union, the biggest portion of food waste originates from households (53%), followed by the sectors of processing (19%), food service (12%), production (11%) and wholesale and retail (5%) [24]. The fact that household food waste (HFW) has a major contribution to the total production of food waste, along with the fact that it is rich in carbon, makes it a valuable organic biomass source for the production of several value-added bioproducts. However, the high-water content, its complex organic matter, the high portion of organic materials being in the solid phase, as well as the variations in HFW composition, make the separate collection and pretreatment of HFW important steps for its successful exploitation.

The aim of this work is to exploit a four air–cathode single chamber MFC design, which is constructed with relatively low-cost materials [25], for bioelectricity production and HFW treatment. This study is the next step of the work [26], where two MFCs were fed with pretreated HFW, and their operation was assessed as a two-unit stack. Specifically, in this study, the cell units were increased in order to treat bigger volumes of waste and generate higher power outputs. In this context, different operation modes were examined. In particular, the performance of four single chamber MFCs, operated with extract from dried and shredded HFW (FORBI), which was selected at the source and collected at municipality level, was assessed. The four units were operated individually and as a stack, in batch and in continuous mode. Both series and parallel electrical configurations were examined. The effect of different operation modes on the microbial fuel cell system performance was evaluated. 

## 2. Results and Discussion

### 2.1. Batch Mode Operation 

**Figure 1:**
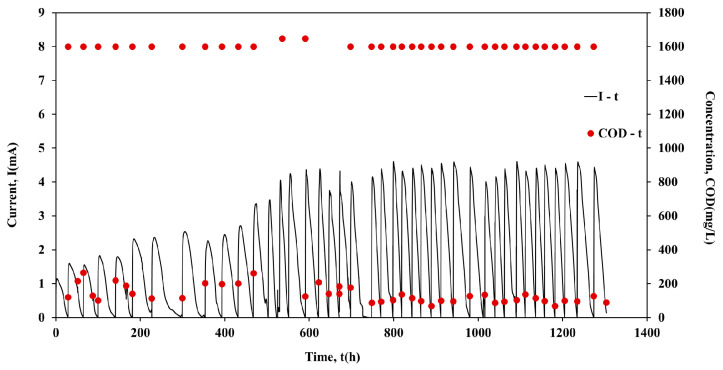
Current output (mA) and COD concentration (mg/L) versus time of Cell 1, during batch operation.

**Figure 2:**
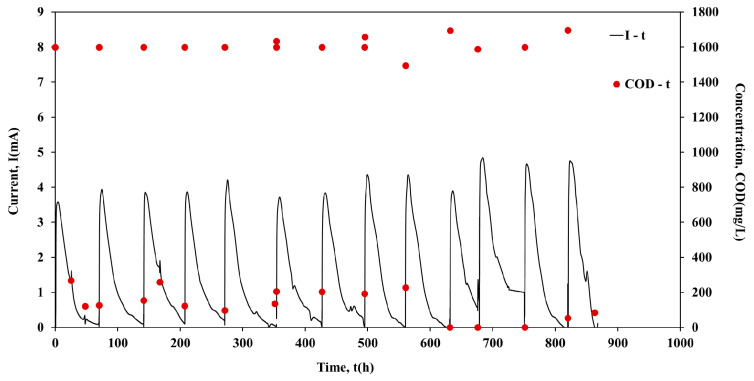
Current output (mA) and COD concentration (mg/L) versus time of Cell 2, during batch operation.

**Figure 3:**
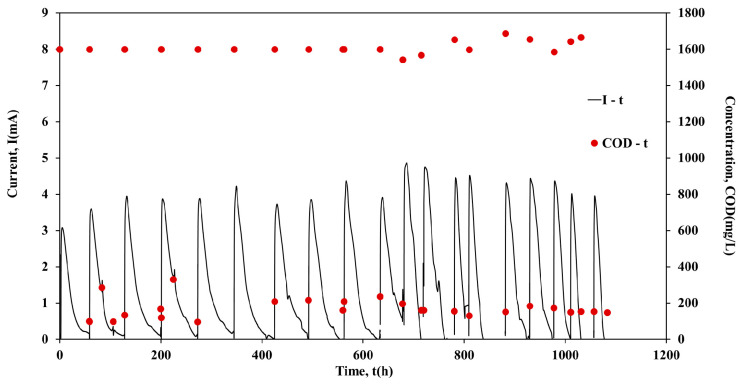
Current output (mA) and COD concentration (mg/L) versus time of Cell 3, during batch operation.

**Figure 4:**
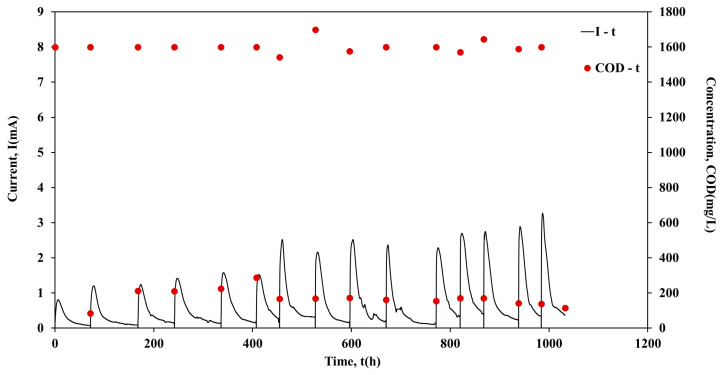
Current output (mA) and COD concentration (mg/L) versus time of Cell 4, during batch operation.

Following the inoculation period, the units operated individually in batch mode for approximately 1000 h. Figure 1, Figure 2, Figure 3 and Figure 4 show the current output and the COD consumption versus time for Cell 1, Cell 2, Cell 3 and Cell 4, respectively. 

As shown in Figure 1, the COD is efficiently removed in all cases (>85%). Moreover, during the first 440 h of Cell 1 operation, the maximum current output (Imax) was in the range of 1.5 mA to 2.5 mA, while the duration of the batch cycles was ~45 h (Figure 2). However, a gradual increase of Imax was observed in the next 150 h of Cell 1 operation. The maximum current output values remained relatively high (4.2 mA to 4.6 mA) for the following ~703 h of Cell 1 operation, while the cycle’s duration decreased to ~25 h. The cycle duration remained almost constant (~70 h) during Cell 2 batch operation, while Imax was in the range 3.6 mA to 4.8 mA, with repeated higher maximum current output values after 685 h of Cell 2 operation (Figure 2). Cell 3 performed similarly to Cell 2, since Imax was in the range of 3.5 mA to 4.8 mA and the duration of the cycles was ~70 h in most of the cycles (Figure 3). On the contrary, although the same conditions and the same handling occurred for Cell 4, Cell 4 underperformed in comparison with Cells 1, 2 and 3. In particular, even though Imax was gradually increased during operation, it remained relatively low within the range of 0.8 mA to 3.3 mA. The duration presented variations among cycles, between the values of 50 h to 100 h (Figure 4). The operation of Cell 4 indicates the difficulty of maintaining similar performance among microbial fuel cells. The units cannot be identical since microbial communities are involved in the process. This is a crucial drawback to overcome, particularly when stacks are constructed and a series connection is used [22].

### 2.2. Continuous Operation

#### 2.2.1. Series Electrical Connection

**Figure 5:**
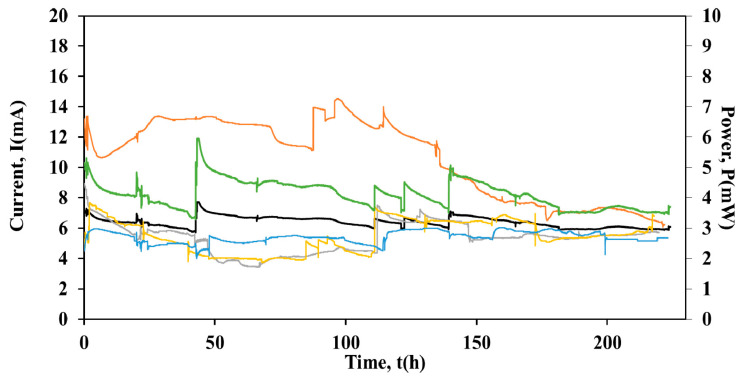
Current output versus time of (a) Cell Stack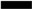, (b) Cell 1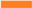, (c) Cell 2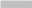, (d) Cell 3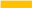, (e) Cell 4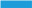 and power versus time of (f) Cell stack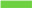, during series connection.

Following the batch operation of the cells, the operation was shifted to continuous mode (48 mL/h). The individual external resistive loads of the units were removed and the four units were connected in series under a common external load of 100 Ω. Figure 5 shows the current and the power output of the stack during time, as well as the current output of the individual units connected in series.

As can be seen from Figure 5, the stack operated for ~222 h under series connection. For the first 154 h of operation, the current output of Cell 1 was I = 12.61 ± 0.96 mA followed by a gradual decrease to 6.24 mA at t = 222 h. The average current output for Cells 2 and 3 and Cell 4 was I = 5.34 ± 0.95 mA, I = 5.50 ± 1.04 mA and I = 5.42 ± 0.38 mA, respectively. Although different cell performances were observed during their batch operation and even though some fluctuations of the current output were observed when the units were serially connected, the stack performance was stable and the voltage reversal phenomenon did not occur. In particular, the average current output of the stack was I = 6.41 ± 0.35 mA, while the average power output was P = 4.1 mW. It is worth mentioning that the higher current output value of Cell 1 gradually decreased close to the respective values of Cells 2, 3 and 4.

Moreover, the COD removal for the flow rate 48 mL/h, was ~67% for Cell 3 and ~58% for Cells 1, 2 and 4, respectively. The high organic consumption during batch operation (COD consumption >85%) indicates that higher organic removal could be achieved at flow rates lower than 48 mL/h. Lower flow rates increase the residence time τ of the anolyte within the anode chamber, thus possibly enhancing the COD consumption efficiency from FORBI extract. 

#### 2.2.2. Parallel Electrical Connection

**Figure 6:**
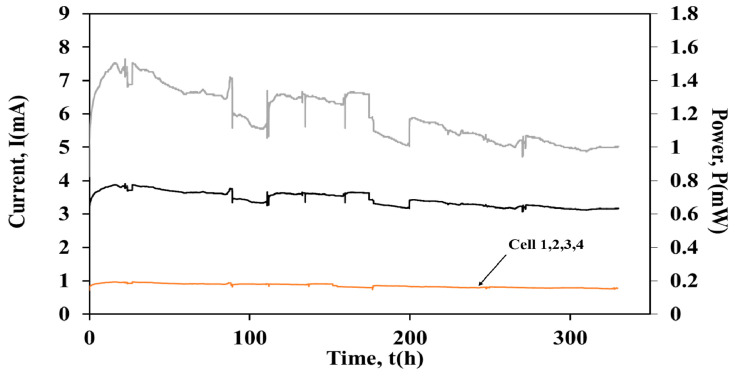
Current output versus time of (a) Cell Stack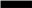, (b) Individual cells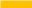 and power versus time of (c) Cell stack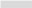, during parallel connection.

Following the series electrical configuration, the units were connected in parallel (Rext = 100 Ω) and the stack operated for ~322 h with this connection. Figure 6 shows the current output of the units and the stack, as well as the power output of the stack in parallel connection.

During the parallel connection no fluctuations were observed on the current output and on the power produced from the stack. Specifically, the average current output was I = 3.43 ± 0.23 mA, while the power was equal to P = 1.2 mW, respectively. The current output of the units was the same (I = 0.86 ± 0.06 mA), while the COD removal was ~62% for Cells 1, 2 and 4 and ~72% for Cell 3.

### 2.3. Electricity Yield during Batch and Continuous Operation 

Table 1 presents the different electricity yields (mJ/gCOD/L) for the cells and the cell-arrays. During batch operation Cells 1, 2 and 3 achieved similar yields, ~20 mJ/gCOD/L, respectively. However, similar to the current output (Figure 5) Cell 4 produced the lowest electricity yield (6 mJ/gCOD/L), when compared to the other three cells. The highest electricity yield (2.6 mJ/gCOD/L) was observed for the stack in continuous operation and series connection. Additionally, the electricity yield for the individual units in series connection was calculated at the 150 h mark (Figure 5), where the current of the individual cells converged to a similar value (~6 mA). The values of the electricity yield were 2 mJ/gCOD/L for Cell 1 and 0.5 mJ/gCOD/L for Cell 2, 3 and 4. 

Furthermore, the electricity yield of the stack in parallel connection was 0.7 mJ/gCOD/L and the electricity yield of the units connected in parallel configuration was ~0.2 mJ/gCOD/L), while in series the yields varied.

It is worth mentioning that although the external resistance of the units and the stacks during batch and continuous mode operation was stable (100 Ω), the external resistance of the units within the stack varied. According to Kirchhoff’s law, the Rext for the individual units in series connection was 25 Ω, while that for the individual units in parallel connection was 400 Ω. Thus, due to the different external resistances, higher current output for the individual units in series connection was observed in comparison with the units in parallel connection (Ohm’s law).

## 3. Materials and Methods

Four identical membraneless, single chamber air–cathode MFCs were constructed, as described in detail in [27]. Each cell consisted of four air–cathode electrodes which ran through the anode chamber. Graphite granules were used as the anodic electrode and MnO_2_ was the cathode catalyst. A graphite-based paste, which contained the cathodic catalyst, was applied on GORE-TEX^®^ cloth. The GORE-TEX^®^ cloth served as the separator and as the catalyst support for each unit [25]. The effective volume of the cells was approximately 120 mL.

The cells were acclimated in batch operation mode, using anaerobic sludge as the inoculum, and glucose (0.8 g COD/L) as the electron donor, similar to [26,27]. An external 100 Ω resistance was connected to each cell. The units were operated at a constant room temperature (25 ± 2 °C).

Following the acclimation period, the synthetic feed was replaced with food residue biomass (FORBI) extract and the units were operated in batch mode for approximately 1000 h. FORBI is a product that originates after the drying and shredding procedure of household food waste (HFW) [6,27,28]. FORBI extract was prepared using the same method described in [28]. Specifically, FORBI was mixed with water and then filtered using a cloth filter. The produced solution was initially filtered with Whatman filters (pore sizes 1.2 μm to 0.7 μm) and then it was diluted to a final concentration of 1.6 gCOD/L. Phosphate buffer was added to the solution (PBS; pH 7) in order to adjust its pH from 3.6 to 7.

**Figure 7:**
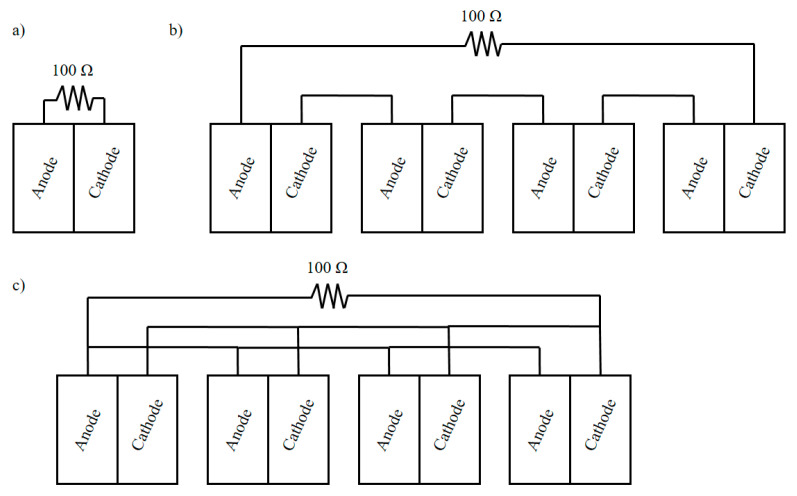
Units’ connection (**a**) in batch mode, (**b**) continuous operation in series connection and (**c**) continuous operation in parallel connection.

During continuous operation the feedstock flowed from the top to the bottom of each unit and went out by overflow, through a constant-level outlet tube, which was placed in the anode chamber. The units were operated at a flow rate 48 mL/h. Each unit was fed individually from a common feeding tank using a peristaltic pump (Masterflex, Cole-Parmer Instrument Company, Vernon Hills, IL, USA). The individual external resistive loads (100 Ω) of the units were removed and the four units were successively connected in series and in parallel connection under a common external load of 100 Ω (Figure 7).

The voltage was recorded for the stacks and the cells individually, at 1 min intervals, using a data acquisition system (Advantech ADAM-4019+). The pH and conductivity were measured by digital instruments (WTW INOLAB PH720) and (WTW INOLAB), respectively. Soluble COD was measured according to the standard methods [29].

The evaluation of the MFC power output was further studied by calculating the electricity yield per gram/L of feed COD. The electricity yield (J/gCOD/L) was calculated by the following Equation (1):

(1) $$El_{Yield}=\frac{\int_{0}^{\Delta t}Pdt}{COD_{FORBI}}$$

where Δ*t* is the duration of the batch cycle, *P* is the power output for the batch cycle and COD_FORBI_ is the inlet COD used in each feed.

In the case of continuous operation the average power output was used instead of the integral, resulting in the following Equation (2):

(2) $$El_{Yield}=\frac{\bar{P}}{COD_{FORBI}}$$

## 4. Conclusions

The work demonstrated that although high COD removals (>85%) were achieved during batch operation, the power output maintained its maximum value (~1.6 mW) per cycle only for ~5 h for each cell. On the other hand, when the units operated continuously, the COD removal was lower (67% to 58% and 62% to 72% for series and parallel connection, respectively) in comparison with batch operation, but a constant power output was obtained (P = 4.1 mW, and P = 1.2 mW for series and parallel connection, respectively). The highest electricity yield (2.6 mJ/gCOD/L) was obtained from the stack connected in series. In addition, although Cell 4 underperformed prior to its series connection, the voltage reversal phenomenon did not occur. This result highlighted the difficulty in setting up identical MFC units, which is an optimal condition before the units are connected in series. However, this was not an issue when the units were electrically connected, indicating the feasibility to further increase the number of units within a stack, thus treating higher volumes of food residue biomass and enhancing the power production.

## Figures and Tables

**Table 1:** The electricity yield (mJ/gCOD/L) of the individual cells and the stacks and the units within the stack during batch and continuous operation.

	Batch	Continuous	
Electricity Yield (mJ/gCOD/L)		Series Connection	Parallel Connection
Cell 1	19	2	0.2
Cell 2	20	0.5	0.2
Cell 3	22	0.5	0.2
Cell 4	6	0.5	0.2
Stack		2.6	0.7

## Data Availability

Not applicable.

## Sample Availability

Samples of the compounds are not available from the authors.
